# Behavior of the Surviving Population of *Listeria monocytogenes* and *Salmonella* Typhimurium Biofilms Following a Direct Helium-Based Cold Atmospheric Plasma Treatment

**DOI:** 10.3389/fmicb.2022.831434

**Published:** 2022-03-24

**Authors:** Marlies Govaert, Cindy Smet, Cyril Acquah, James L. Walsh, Jan F. M. Van Impe

**Affiliations:** ^1^CPMF2 - Flemish Cluster Predictive Microbiology in Foods, Ghent, Belgium; ^2^OPTEC - Optimization in Engineering Center-of-Excellence, KU Leuven, Ghent, Belgium; ^3^BioTeC+ - Chemical and Biochemical Process Technology and Control, Department of Chemical Engineering, KU Leuven, Ghent, Belgium; ^4^Department of Electrical Engineering and Electronics, University of Liverpool, Liverpool, United Kingdom

**Keywords:** cold atmospheric plasma, biofilms, surviving population, regrowth, *Listeria monocytogenes*, *Salmonella* Typhimurium, resistance and susceptibility

## Abstract

Although the Cold Atmospheric Plasma (CAP) technology proved promising for inactivation of biofilms present on abiotic food contact surfaces, more research is required to examine the behavior of the CAP surviving biofilm-associated cells. It was therefore examined whether (i) CAP treated (*Listeria monocytogenes* and *Salmonella* Typhimurium) biofilm-associated cells were able to further colonize the already established biofilms during a subsequent incubation period and (ii) isolates of the surviving population became less susceptible toward CAP when the number of biofilm development—CAP treatment cycles increased. For this purpose, a direct treatment was applied using a helium-based Dielectric Barrier Discharge electrode configuration. Results indicated that the surviving population was able to further colonize the already established biofilms, since the cell density of the CAP treated + incubated biofilms equaled the initial density of the untreated biofilms. For the *L. monocytogenes* biofilms, also the total biomass proved to further increase, which might result in an even further increased resistance. The susceptibility of the biofilm-associated cells proved to be influenced by the specific number of CAP treatment cycles, which might potentially result in an overestimation of the CAP treatment efficacy and, consequently, an increased risk of food contamination.

## Introduction

Biofilms are functional consortia of cells which are protected by a matrix of Extracellular Polymeric Substances (EPS). These matrix compounds have been produced by the cells themselves and enable them to remain attached to each other and/or (a)biotic surfaces (Bakke et al., 1984; Costerton et al., 1987; Kumar and Anand, 1998; Jefferson, 2004; Garrett et al., 2008; Giaouris et al., 2014). In addition, this protective layer also results in a better retention of water and nutrients and a limited diffusion of antimicrobial agents and antibiotics into the biofilm. These characteristics are, among others, responsible for the high resistance of biofilm-associated cells toward different (traditional) biofilm inactivation methods such as the use of hot water in combination with antimicrobial agents and a mechanical action (Kumar and Anand, 1998; Costerton et al., 1999; Ciofu and Tolker-Nielsen, 2011; Gómez-López, 2012).

In more recent studies, novel technologies have been examined for their ability to inactivate biofilms. One of these novel technologies is the use of Cold Atmospheric Plasma (CAP). This specific type of plasma can be generated by means of the addition of energy to a gas at atmospheric pressure and room temperature. As a result, a variety of reactive species is generated, including ions, photons, charged particles, free electrons, and radicals (Tendero et al., 2006; Misra et al., 2011; Banu et al., 2012; Fernández and Thompson, 2012; Patil et al., 2016). The CAP technology already proved to be able to inactivate biofilms developed by different (pathogenic) species such as *Listeria monocytogenes*, *Salmonella* Typhimurium, *Escherichia coli*, *Candida albicans*, *Staphylococcus epidermidis*, and *Pseudomonas aeruginosa* (e.g., Maisch et al., 2012; Niemira et al., 2014; Ziuzina et al., 2014, 2015; Govaert et al., 2019a; Modic et al., 2019). For *L. monocytogenes* and *S.* Typhimurium in specific, the target species of this research, inactivation of both single- and dual-species model biofilms was previously obtained using a (direct) helium-based Dielectric Barrier Discharge (DBD) electrode with a dissipated plasma power of approximately 7.0 W (Govaert et al., 2018b, 2019a,b). However, complete inactivation of the biofilms was not achieved, i.e., a subpopulation of the biofilm-associated cells proved to be resistant toward the applied CAP treatment. The behavior of this surviving population should be investigated in order to decide whether the CAP technology can be safely implemented in the food industry for inactivation of biofilms grown on abiotic food contact surfaces. In this regard, two important aspects need to be considered, i.e., whether these cells are able to (i) further colonize the existing biofilm by continuing to multiply and/or form EPS and (ii) form new biofilms, with a potentially increased resistance or lowered susceptibility toward CAP, elsewhere on the surface.

If the surviving population of biofilm-associated cells is able to continue multiplying within the already established biofilm, the effect of the CAP treatment will eventually be nullified. In addition, if these surviving biofilm-associated cells are able to continue producing EPS, the protective effect of the matrix compounds can potentially contribute to an increased resistance or lowered susceptibility of the cells toward a consecutive (CAP) treatment. Moreover, the study of Müsken et al. (2018), which was performed on clinical biofilms, indicated that the increased production of EPS might result in a strong inflammatory infection as a result of the accumulation of biofilm debris. Nevertheless, previously performed studies (e.g., Müsken et al., 2018; Ricker et al., 2018; Han et al., 2019; Zhang et al., 2019) mainly focused on the viable cell density of the recovered biofilms, while ignoring this potentially increased production of EPS. In addition, these inactivation studies were mainly performed using traditional inactivation methods such as the use of antimicrobial agents and antibiotics. Based on these studies, it can, however, be concluded that the applied treatment (type, efficacy, concentration, and time) and the environmental conditions encountered during subsequent incubation exert a significant influence on the ability of the surviving biofilm-associated cells to regrow. The study of Ricker et al. (2018), for example, reported that *P. aeruginosa* biofilms were not able to regrow following thermal inactivation when the applied thermal shock was able to reduce the viable cell density below 10^3^ CFU/cm^2^. The research of Han et al. (2019), on the other hand, treated saliva-based biofilms with different antimicrobial agents and indicated that the initial biofilm cell density was restored at a higher rate when the interval between two medium refreshment steps was reduced. So far, only two previous studies specifically focused on the ability of CAP treated biofilm-associated cells to regrow when the treated biofilms were further incubated at biofilm-promoting environmental conditions. Traba and Liang (2015), on the one hand, used an argon-based plasma system for inactivation of *Staphylococcus aureus* biofilms which were afterward incubated for up to 150 h with fresh growth medium. The results indicated that these biofilms were indeed able to regrow since the total biomass of the CAP treated biofilms increased as function of the incubation time. Modic et al. (2019), on the other hand, observed that regrowth of biofilm-associated *E. coli* and *S. epidermidis* cells depended on the applied CAP treatment characteristics. Two different air-based plasma systems were used, i.e., an indirect Surface Barrier Discharge (SBD) electrode and a direct plasma jet. Using the former set-up, the CAP surviving population of biofilm-associated cells was able to regrow until a similar cell density as for the untreated biofilms. Using the latter set-up, however, biofilm regrowth was inhibited.

Previous studies also reported that CAP treatment can potentially result in dispersal of (some parts of) the biofilm (e.g., Traba and Liang, 2011; Mai-Prochnow et al., 2016; Gilmore et al., 2018). When this phenomenon occurs, the surviving population of biofilm-associated cells can become planktonic again and start to form a biofilm elsewhere on the surface (provided that they are still able to multiply and produce matrix compounds; Kumar and Anand, 1998). Later on, these newly developed biofilms can again be exposed to CAP (or any other inactivation method), so it is of high importance to examine whether the resistance/susceptibility of these biofilms toward CAP treatment is affected by the number of consecutive biofilm development—CAP treatment cycles. To the best of the authors’ knowledge, this has never been examined before. However, similar studies on clinical biofilms indicated that these biofilms indeed become more resistant toward antibiotics when they are exposed to sub-lethal concentration (e.g., Ahmed et al., 2018). The Centers for Disease Control and Prevention (2020) mentions that this resistance build-up is caused by the survival of a subpopulation of antibiotic-resistant cells which are consequently able to (i) continue multiplying and/or (ii) directly transfer their resistance genes to other non-resistant cells/species. For biofilm-associated cells in specific, these aspects can be facilitated by (i) the limited penetration of antimicrobial agents into the EPS matrix, resulting in a high amount of cells which are only exposed to sub-lethal concentrations, (ii) the close proximity of the cells, and (iii) the extracellular DNA present within the EPS matrix (Costerton et al., 1987; Kumar and Anand, 1998; Bridier et al., 2011). If a similar resistance build-up (or a lowered susceptibility) would occur following CAP treatment of biofilms, additional measures should be taken to prevent further biofilm formation and/or to further inactivate the surviving biofilm-associated cells.

Within this study, it was therefore examined whether (i) the surviving population of CAP treated biofilms was able to continue multiplying and/or forming EPS and (ii) CAP treated biofilm-associated cells became more resistant or less susceptible as the number of consecutive biofilm development—CAP treatment cycles increased. These phenomena were investigated for two single-species model biofilms developed by *L. monocytogenes* (Gram positive) and *S.* Typhimurium (Gram negative) cells and a direct helium-based CAP treatment was applied using a DBD electrode configuration. These pathogenic species are highly relevant for the food industry due to (i) their ability to form biofilms on food contact surfaces and (ii) the potentially severe foodborne illnesses associated with their occurrence in/on contaminated food products.

## Materials and Methods

### Experimental Design

**Figure 1:**
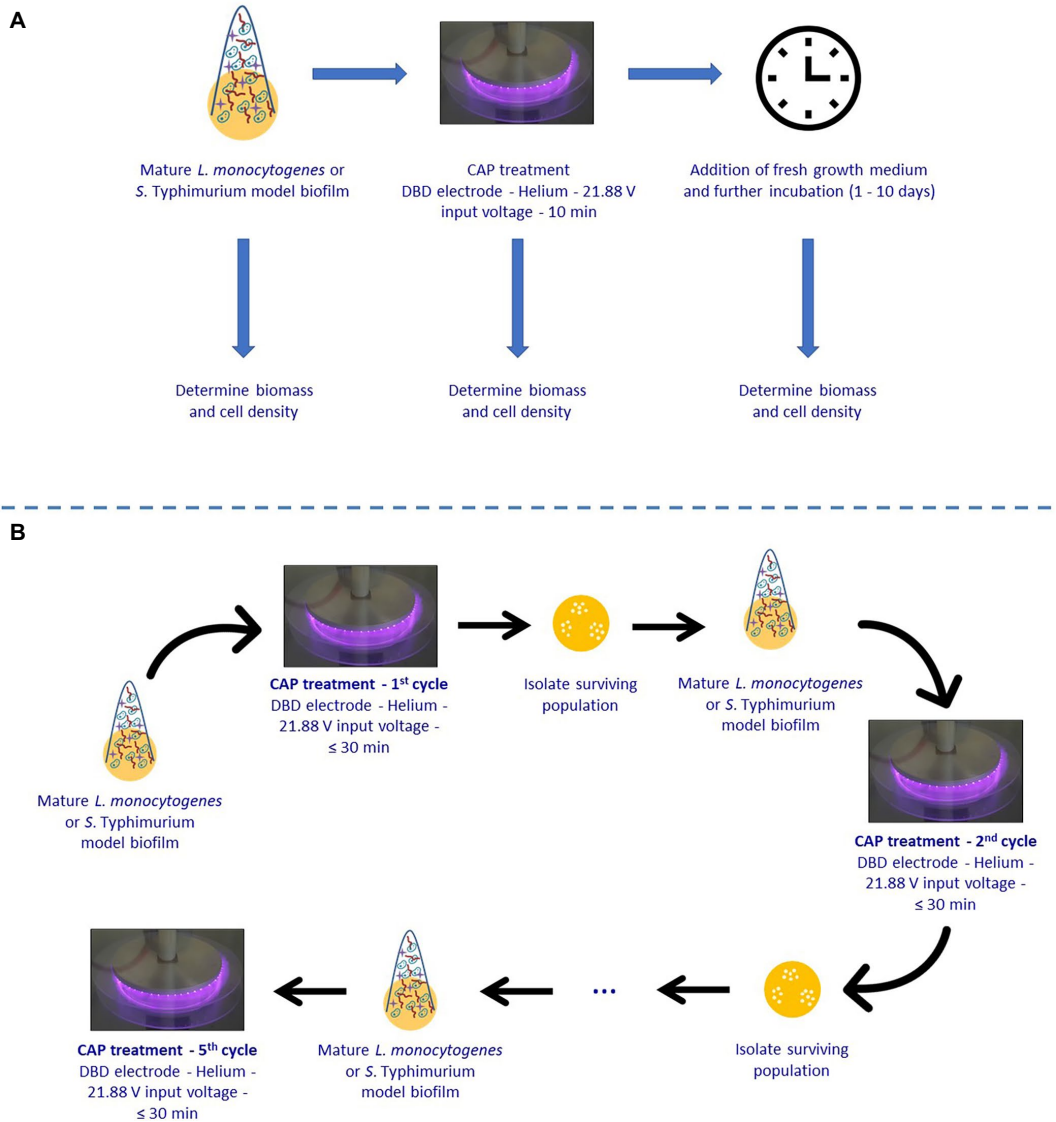
Experimental design used within the presented study. **(A)** Ability of surviving Cold Atmospheric Plasma (CAP) treated biofilm-associated cells to continue multiplying and/or forming Extracellular Polymeric Substances (EPS). **(B)** Susceptibility of biofilm-associated cells toward consecutive CAP treatments.

Within this research, the experimental design illustrated in Figure 1 has been used. In the first part of this study, single-species *L. monocytogenes* and *S.* Typhimurium model biofilms were developed and CAP treated for 10 min using previously determined optimal CAP treatment conditions (Govaert et al., 2019a). Around 10 min was selected, as a further extension of CAP treatment time did not result in additional inactivation (Govaert et al., 2019a). Afterward, fresh (optimal) growth medium was added and the biofilms were further incubated for 1, 2, 3, 7, or 10 day(s) at previously determined optimal biofilm formation temperatures (Govaert et al., 2018a). It was opted to add fresh growth medium in order to mimic food handling which restarts immediately following decontamination of the CAP treated food contact surfaces. Finally, both the cell density and the total biomass of the untreated, CAP treated, and CAP treated + incubated biofilms were determined in order to examine whether the surviving biofilm-associated cells were able to continue multiplying and/or producing EPS. In order to do this, the cell density and total biomass were determined by means of viable plate counts and optical density (OD) measurements following crystal violet staining, respectively.

In the second part of this research, it was examined whether the surviving biofilm-associated cells can become more resistant or less susceptible to CAP when they are subjected to a number of consecutive CAP treatments. For this purpose, single-species *L. monocytogenes* and *S.* Typhimurium model biofilms were CAP treated for different treatment times ranging between 0 and 30 min using previously determined optimal CAP treatment conditions (cycle x). Isolates obtained from the 30 min samples were subsequently used to develop new mature biofilms, which were again CAP treated for up to 30 min in a next cycle (cycle x + 1). This procedure was several times repeated in order to subject the biofilm-associated cells to five consecutive CAP treatment cycles. For each cycle, the viable plate counting method was used in combination with predictive models (i.e., the model of Geeraerd et al., 2000) to determine the inactivation kinetics.

### Microorganisms, Pre-culture Conditions, and Biofilm Development Conditions

Within this study, single-species model biofilms were developed for two bacterial species, i.e., *L. monocytogenes* LMG23775 (isolated from sausages) and *S.* Typhimurium LMG14933 (isolated from bovine liver). For both species, freeze-dried cultures were acquired from the BCCM/LMG bacteria collection of Ghent University in Belgium and stock-cultures were prepared according to the provided protocol. For *L. monocytogenes*, on the one hand, stock-cultures were prepared in Brain Heart Infusion broth (BHI, VWR International, Belgium) which was supplemented with 20% (v/v) glycerol (VWR International, Belgium). For *S.* Typhimurium, on the other hand, stock-cultures were prepared in Tryptic Soy Broth (TSB, Becton Dickinson, United States) which was also supplemented with 20% (v/v) glycerol.

At the start of each experiment, a purity plate was prepared by spreading a loopful of stock-culture into a LB agar plate [Lennox Luria Bertani agar (Becton Dickinson, United States)] supplemented with 5 g/L NaCl (Sigma-Aldrich, United States). The purity plates for *L. monocytogenes* and *S.* Typhimurium were incubated for 24 h at 30 and 37°C, respectively. Starting from these purity plates, pre-cultures were prepared by transferring one colony into an Erlenmeyer flask containing 20 ml of LB medium [Lennox Luria Bertani broth (Becton Dickinson, United States) supplemented with 5 g/L NaCl]. *Listeria monocytogenes* and *S.* Typhimurium pre-cultures were incubated for 24 h at 30 and 37°C, respectively. Following this incubation period, stationary phase cultures with a cell density of approximately 10^9^ CFU/ml were obtained. These cultures were used to develop a 100-fold diluted inoculum with a cell density of approximately 10^7^ CFU/ml. BHI and 20-fold diluted TSB (TSB/20) were used as dilution media for *L. monocytogenes* and *S.* Typhimurium, respectively, since these growth media proved to be optimal for their biofilm formation (Govaert et al., 2018a).

The inoculum was then transferred to small Petri dishes made out of polystyrene (50 mm diameter and 9 mm height, Simport, Canada). For each Petri dish/biofilm, 1.2 ml of inoculum was required and the Petri dishes were gently shaken to make sure the inoculum covered the entire surface. Finally, the closed Petri dishes were incubated for 24 h at 25 (*S*. Typhimurium) or 30°C (*L. monocytogenes*), which proved to be the optimal temperatures for biofilm formation (Govaert et al., 2018a).

Within the first part of this research, previously explained procedure was repeated for each model biofilm. In other words, the same protocol was followed for the untreated, CAP treated, and CAP treated + incubated *L. monocytogenes* and *S.* Typhimurium model biofilms. Within the second part of this research, purity plates were only required for the first CAP treatment cycle. For all other CAP treatment cycles, isolates from the previous cycle were used to make pre-cultures and model biofilms. More specifically, these isolates were obtained by plating aliquots of the (diluted) 30 min CAP treated samples on non-selective medium (see Section “Quantification of the Biofilm Cell Density by Means of Viable Plate Counts and Procedure Used to Collect Isolates”). The pre-culture conditions and model biofilm development procedure were, however, the same as for the first part of this study.

### CAP Equipment and Biofilm Inactivation Procedure

Optimal CAP treatment conditions involved (i) the use of a direct Dielectric Barrier Discharge (DBD) electrode configuration, (ii) helium as working gas (purity 99.996%; flow rate 4 L/min), and (iii) an input voltage of 21.88 V (resulting in a high-voltage signal of approximately 6.5 kV and a dissipated plasma power of approximately 7.0 W). The authors refer to Govaert et al. (2019a) for a detailed overview of the characteristics of the applied DBD electrode configuration.

Prior to CAP treatment, the biofilms were removed from the incubator and three times rinsed with phosphate buffered saline (PBS) solution to remove the remaining planktonic cells. After this rinsing procedure, the biofilms were allowed to dry in the laminar flow cabinet for approximately 20 min. The rinsed and dried Petri dishes were then placed in between the electrodes and the reactor chamber was flushed for 4 min to ensure a homogeneous gas mixture. Finally, the high-voltage power source was energized to generate the plasma. Samples were treated for up to 30 min and immediately after the treatment removed from the reactor chamber to (i) add fresh growth medium prior to further incubation (part 1), (ii) quantify the total biomass or the remaining viable cell density (see Sections “Quantification of the Total Biomass by Means of Crystal Violet Staining” and “Quantification of the Biofilm Cell Density by Means of viable Plate Counts and Procedure Used to Collect Isolates”; part 1 and part 2), or (iii) collect isolates from the surviving population of biofilm-associated cells (see Section “Quantification of the Biofilm Cell Density by Means of viable Plate Counts and Procedure Used to Collect Isolates”; part 2).

### Quantification of the Total Biomass by Means of Crystal Violet Staining

The method used for quantification of the total biomass of the (un)treated (and further incubated) model biofilms has been discussed in detail in the research of Govaert et al. (2018a). In brief, the different steps of this assay were as: (i) fixation of the rinsed (un)treated (and further incubated) biofilms with methanol [99% (v/v), VWR Chemicals, Belgium], (ii) staining with a 2% (v/v) crystal violet solution (Sigma-Aldrich, United States), (iii) removal of excess stain, (iv) addition of glacial acetic acid solution [33% (v/v), VWR International, Belgium] to redissolve the remaining stain, and (v) OD measurement at 570 nm using a VersaMax tunable microplate reader (Molecular devices, United Kingdom). If the OD was higher than 1, the solution was diluted using the glacial acetic acid solution and a correction factor was incorporated to determine the OD of the original solution.

### Quantification of the Biofilm Cell Density by Means of Viable Plate Counts and Procedure Used to Collect Isolates

Throughout this research, the (remaining) cell density of the (un)treated (and further incubated) model biofilms was determined by means of viable plate counts. For this purpose, 2 ml of sterile PBS solution was added to the rinsed and dried (un)treated (and further incubated) biofilms and a cell scraper (blade width 20 mm, Carl Roth GmbH+Co, Germany) was used to remove the biofilms from the surface. Serial decimal dilutions of the obtained cell suspensions were prepared [0.85% (v/v) NaCl] and plated on agar plates. For each of the serial dilutions, three drops of 20 μl were plated on non-selective and selective media. Brain Heart Infusion agar (BHIA, BHI supplemented with 14 g/L biological agar, VWR Chemicals, Belgium) was used as non-selective medium for *L. monocytogenes*, while Tryptic Soy Agar (TSA, TSB supplemented with 14 g/L bacteriological agar) was used for *S.* Typhimurium. PALCAM (VWR Chemicals, Belgium) and XLD (Xylose Lysine Deoxycholate, VWR Chemicals, Belgium) were used as selective media for *L. monocytogenes* and *S.* Typhimurium, respectively. This was done in order to diversify subpopulations of CAP treated cells: (i) dead/inactivated cells for which the applied treatment was lethal, (ii) healthy cells where the applied treatment did not have any effect, and finally (iii) sub-lethally injured (SI) cells, which are damaged but not killed (Hurst, 1977; Wu and Fung, 2001). Both healthy and injured cells are able to form colonies on the non-selective media, while the selective media only enable growth of healthy cells (Noriega et al., 2013). Based on the difference in colony forming units (CFU) obtained using both media, it is possible to examine whether the applied CAP treatment can induce sub-lethal injury of the biofilm-associated cells (see Section “Modeling and Parameter Estimation”). Before counting the colonies, agar plates were incubated for (at least) 24 h at 30 (BHIA and PALCAM) or 37°C (TSA and XLD) and the detection limit of the applied plate counting method was 1.2 log10 (CFU/cm^2^).

Within the second part of this study, isolates from the 30 min CAP treated samples were collected by plating three drops of 20 μl of dilutions 100, 10^−1^, and 10^−2^ on the appropriate non-selective medium to have at least one dilution resulting in the formation of non-clustered colonies. Following 24 h of incubation at 30 (BHI, *L. monocytogenes*) or 37°C (TSA, *S.* Typhimurium), these plates were stored in the fridge to make pre-cultures for the subsequent CAP treatment cycle (see Section “Microorganisms, Pre-culture Conditions, and Biofilm Development Conditions).

### Modeling and Parameter Estimation

Within the first part of this study, the percentage of sub-lethally injured cells (% SI) present within the untreated, CAP treated, and CAP treated + incubated model biofilms was determined using both the non-selective and the selective medium counts in combination with the equation of Busch and Donnelly (1992) (Equation 1).


(1) $$\%\mathrm{SI}=\frac{\mathrm{CFU}\;non-selectivemedium-\mathrm{CFU}\;selectivemedium}{\mathrm{CFU}\;non-selectivemedium}\times 100$$

Within the second part of this research, the model of Geeraerd et al. (2000), describing a microbial inactivation curve consisting of a log-linear inactivation phase and a tail (Equation 2), was used to fit the experimental data obtained following CAP treatment of the biofilms.


(2) $$N\left(t\right)=\left(N_{0}-N_{res}\right)\cdot e^{-k_{max}\cdot t}+N_{res}$$

Here, *N(t)* [CFU/cm^2^] is the cell density at time *t* [min], *N_0_* [CFU/cm^2^] is the initial cell density, *N_res_* [CFU/cm^2^] is a more resistant subpopulation, and *k_max_* [1/min] is the maximum specific inactivation rate. The log-transformed version of the above model was used in calculations. Based on the difference between log *N_0_* and log10 *N_res_*, the final log10-reduction values (following 30 min of CAP treatment) were calculated.

The parameters of the Geeraerd et al. (2000) model were estimated *via* the minimization of the sum of squared errors (SSE), using the lsqnonlin routine of the Optimization Toolbox of Matlab version R2016a (The Mathworks, Inc.). At the same time, SEs of the parameter estimations were determined based on the Jacobian matrix. The Root Mean Squared Error (RMSE) served as an absolute measure of the goodness of the model to fit the actual obtained data.

To calculate the percentage of sub-lethally injured cells (% SI) as function of the CAP treatment time, the equation of Busch and Donnelly (1992) was again used. However, as compared to the first part of this study, this percentage was not calculated based on the real data points but based on the theoretical concentrations obtained from the Geeraerd et al. (2000) model. For each CAP treatment time (0–30 min), the percentage of sub-lethally injured cells was thus determined based on the value of the model fit for both the non-selective and the selective medium.

### Statistical Analysis

In the first part of this study, ANOVA tests were performed to determine whether there were any significant differences among means of OD values or logarithmically transformed viable counts obtained for the untreated, CAP treated, and CAP treated + incubated model biofilms. For the biomass experiments, on the one hand, at least five independent biological replicates were used for each condition (i.e., untreated, CAP treated, and CAP treated + incubated). For the viable cell density determination, on the other hand, at least three independent biological replicates were used.

In the second part of this research, ANOVA tests were again applied to determine whether there were significant differences between the estimated model parameters obtained for the different CAP treatment cycles. Separate ANOVA tests were performed for each (i) model parameter, (ii) biofilm forming species (*L. monocytogenes* and *S.* Typhimurium), and (iii) type of medium (non-selective and selective). For each CAP treatment cycle, data points were collected at 10 different treatment times using at least two independent biological replicates.

All statistical analyses were performed using Matlab version R2016a (The Mathworks, Inc.). A confidence level of 95.0% (*α* = 0.05) was applied and Fisher’s Least Significant Difference (LSD) test was used to distinguish which means were significantly different from others. Significant differences between sample values/estimated model parameters were indicated with different (uppercase) letters (e.g., “a,” “b,” “A,” and “B”), with “a” and “A” indicating the lowest value.

## Results and Discussion

### Ability of the Surviving CAP Treated Biofilm-Associated Cells to Continue Multiplying and/or Forming EPS

**Figure 2:**
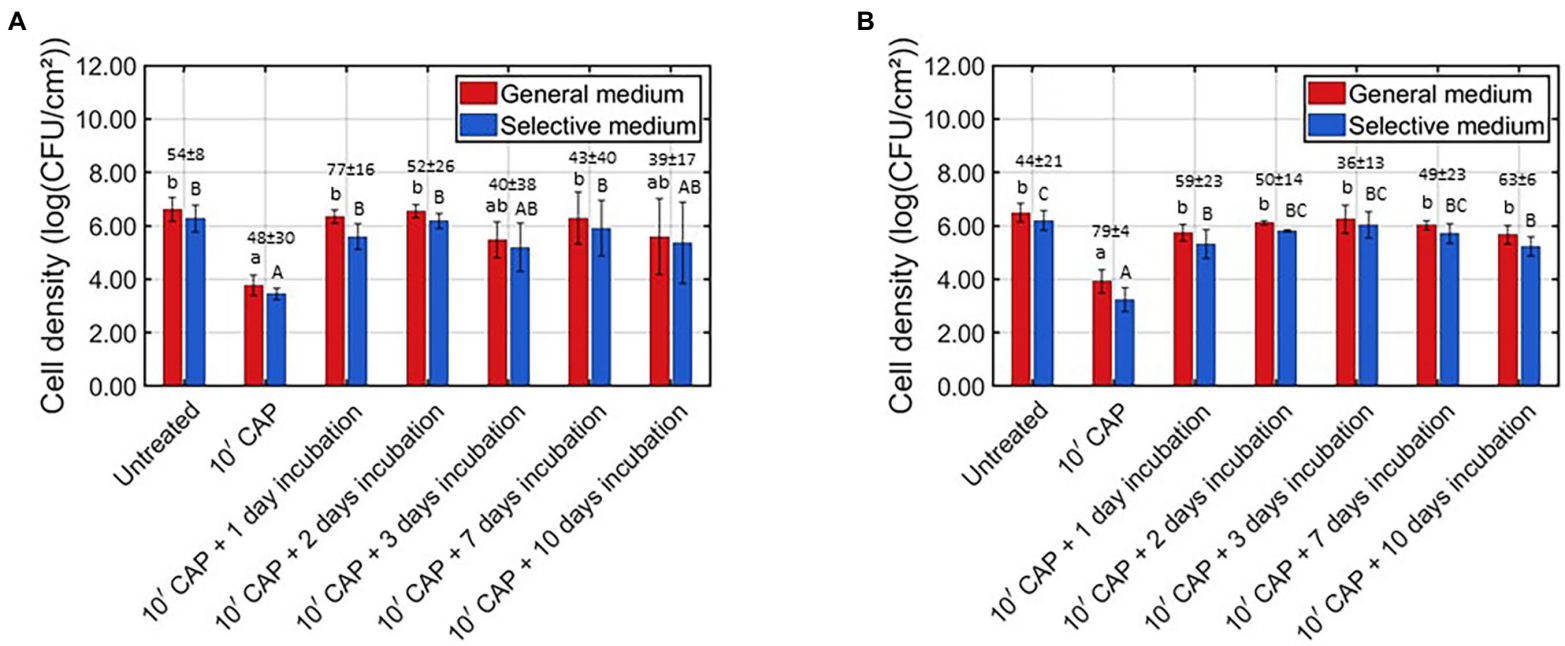
Viable cell density [log (CFU/cm^2^)] of the untreated, CAP treated, and CAP treated + incubated model biofilms determined on non-selective and selective medium (*n* = 3). Moreover, the corresponding sub-lethal injury values (average ± stdev) have been presented at the top of the bars. **(A)** *Listeria monocytogenes* model biofilms and **(B)** *Salmonella* Typhimurium model biofilms.

**Figure 3:**
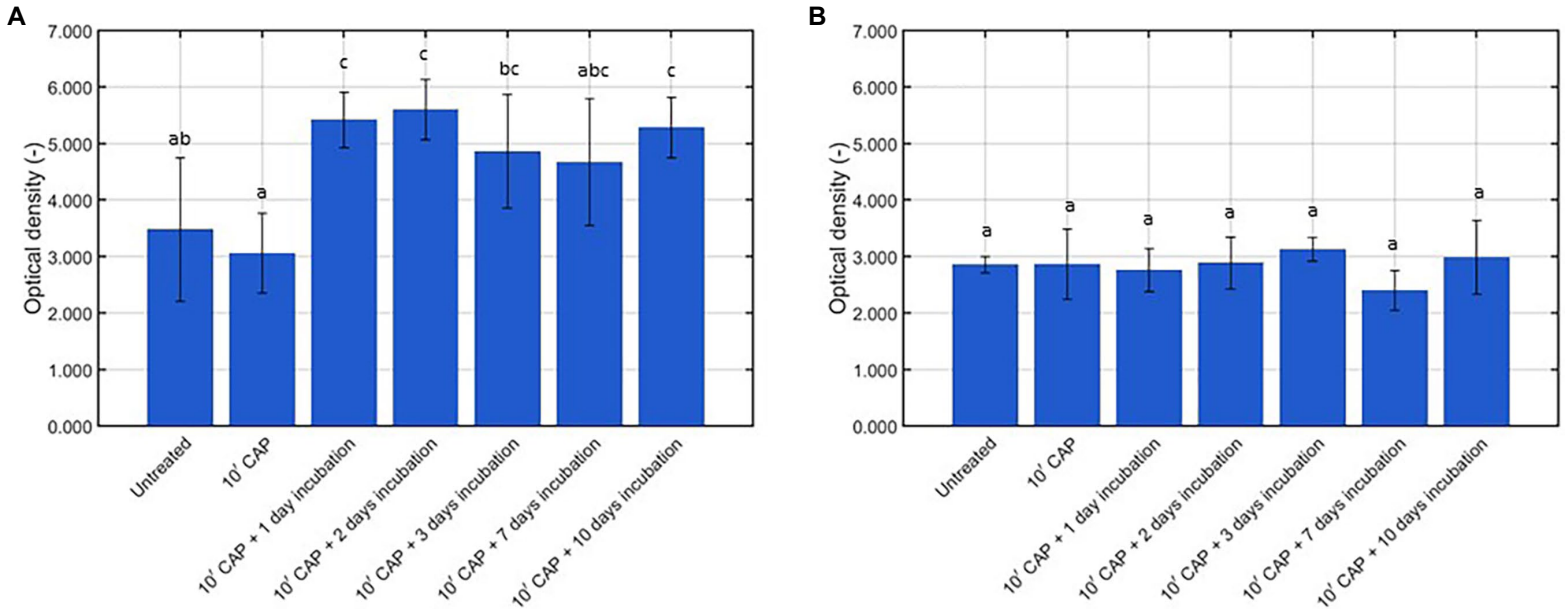
Optical density (OD; -) following crystal violet staining of the untreated, CAP treated, and CAP treated + incubated model biofilms (*n* = 5). **(A)** *Listeria monocytogenes* model biofilms and **(B)** *Salmonella* Typhimurium model biofilms.

As mentioned before, the behavior of the surviving CAP treated biofilm-associated cells was characterized based on their ability to continue (i) multiplying and (ii) forming EPS. For this purpose, the cell density and the total biomass were determined (i) for the untreated model biofilms, (ii) following 10 min of CAP treatment, and (iii) following 10 min of CAP treatment and further incubation for 1–10 day(s). For *L. monocytogenes*, on the one hand, the results obtained for the cell density and the total biomass were presented in Figures 2A, 3A, respectively. For *S.* Typhimurium, on the other hand, these respective results were presented in Figures 2B, 3B. For each of the examined conditions, both the average percentage of sub-lethally injured cells and the corresponding standard deviation have been presented at the top of the bars in Figure 2A (*L. monocytogenes*) and Figure 2B (*S.* Typhimurium).

#### *Listeria monocytogenes* Model Biofilms

Based on Figure 2A, it was observed that the viable cell density significantly reduced following 10 min of CAP treatment, i.e., the average viable cell density decreased from approximately 6.5 log_10_ (CFU/cm^2^) to approximately 3.7 log_10_ (CFU/cm^2^). Similar promising log_10_-reduction values were observed by Govaert et al. (2019a) using the same CAP treatment procedure. Nevertheless, when fresh growth medium was added to the CAP treated biofilms, 1 day of incubation at the optimal biofilm formation temperature was already sufficient to reach a similar viable cell density as for the untreated *L. monocytogenes* model biofilms. When the CAP treated biofilms were incubated for an even longer period of time (i.e., up to 10 days), there was no further change of the viable cell density as compared to the values obtained following 1 day of incubation. In the research of Traba and Liang (2015), examining regrowth of *S. aureus* biofilms following treatment with an argon-based plasma system and subsequent addition of fresh growth medium (i.e., TSB supplemented with 0.2% glucose), similar results were obtained as within the presented study, i.e., the cell density of the CAP treated biofilms increased as function of the incubation time. Nevertheless, these conclusions were not drawn based on viable plate counts, but on OD measurements which do not enable the detection of a relatively small increase of the viable cell density. Moreover, the obtained OD values were only compared with the corresponding values obtained following CAP treatment and not with those obtained for the untreated biofilms. Modic et al. (2019) previously examined whether CAP treated *E. coli* and *S. epidermidis* biofilm-associated cells were able to regrow following an incubation period of 24 h at optimal biofilm formation conditions. The results indicated that regrowth depended on the applied CAP characteristics. Regrowth was observed for an indirect air-based treatment (using a SBD electrode configuration), but not for the corresponding direct treatment (using a plasma jet). This was deemed to be a consequence of the different reactive plasma species, which were generated and the ability of these species to interact with the biofilm-associated cells. Nevertheless, the direct treatment applied within the presented study still resulted in regrowth of the biofilm-associated cells, which is most likely also a consequence of the different operating conditions which have been applied. For example, the operating gas (helium vs. air) mainly determines which plasma species are generated. Therefore, one should be careful with extrapolating the findings of one study, using a specific plasma system, to another.

Similar viable cell density results were observed for both the non-selective and the selective medium, although generally lower values were always obtained using the selective medium (Figure 2A). Consequently, it can be concluded that each examined condition resulted in sub-lethal injury of the biofilm-associated cells. When comparing the sub-lethal injury percentages, it was observed that the highest (average) value was obtained when the *L. monocytogenes* model biofilms were CAP treated and further incubated for 1 day. For this specific condition, on average 77% of the biofilm-associated cells were sub-lethally injured, while this was only the case for 54% of the untreated and 48% of the CAP treated biofilm-associated cells. The effect of the CAP treatment was thus not only nullified following 1 day of incubation, the (average) percentage of sub-lethally injured cells even further increased as compared to the (high) initial percentage obtained for the untreated model biofilms. This is of high importance since this might cause health risks due to an underestimation of the level of contamination when only selective media are used during microbial assessment of food contact surfaces (Noriega et al., 2013).

With respect to the total biomass of the *L. monocytogenes* model biofilms (Figure 3A), it can be concluded that 10 min of CAP treatment did not result in a reduced biomass as compared to the untreated model biofilms. This has been observed before in the research of Govaert et al. (2019c), i.e., 10 min of CAP treatment using the optimal CAP treatment conditions did not result in any (significant) removal of the *L. monocytogenes* and *S.* Typhimurium model biofilm due to the lack of an etching effect. Following further incubation of the CAP treated model biofilms; however, an increased total biomass was observed as compared to the untreated (and CAP treated) model biofilms. Significant differences were, however, only detected following 1, 2, and 10 day(s) of incubation. This increase in total biomass can be the result of (i) the observed increase in viable cell density (Figure 2A) and/or (ii) an increased production of matrix compounds. In a previous study of Govaert et al. (2018a), the crystal violet assay proved to be unable to detect a similar increase in viable cell density due to the low sensitivity of this quantification method. Therefore, it can be concluded that the increase in biomass was (mainly) the result of an increased production of matrix compounds. As for the cell density results, similar results were observed by Traba and Liang (2015) following CAP treatment of the *S. aureus* biofilms, i.e., the total biomass determined following crystal violet staining of the biofilms increased as function of the incubation time.

Based on aforementioned results, it can be concluded that the CAP treated *L. monocytogenes* biofilm-associated cells were still able to (i) continue multiplying and (ii) produce EPS. As a result, the effect of the CAP treatment was already nullified following 1 day of incubation at the optimal biofilm formation conditions. Moreover, these newly established model biofilms potentially became even more resistant (or less susceptible) toward CAP or any other inactivation method due to the even further increased production of EPS. Many previous studies indicated that the EPS matrix is one of the main attributes responsible for the high resistance of biofilm-associated cells (Costerton et al., 1987; Kumar and Anand, 1998). For the specifically used *L. monocytogenes* model biofilms, the increased resistance at an increased total biomass has been confirmed before by the research of Govaert et al. (2018b). Untreated *L. monocytogenes* model biofilms were incubated for up to 10 days and both the total biomass and the resistance toward CAP were determined at different incubation times. The results indicated that the total biomass of the *L. monocytogenes* biofilms significantly increased starting from 7 days of incubation. For these 7 days old model biofilms, this also resulted in an increased resistance of the biofilm-associated cells toward CAP (Govaert et al., 2018b). Another aspect that indicates a potentially increased resistance of the newly established biofilms is the fact that the viable cell density was the same for each incubation time. This is in contradiction to the study of Govaert et al. (2018b), where the cell density of untreated *L. monocytogenes* biofilms proved to significantly decrease following 7 and 10 days of incubation, most likely due to nutrient depletion and/or waste accumulation. Based on this, it can be hypothesized that the specifically applied CAP treatment resulted in some phenotypical changes and/or the selection of a more resistant subpopulation of cells, making these cells more resistant/less susceptible toward stressful environmental conditions. However, this phenomenon needs to be examined in more detail in future studies.

#### *Salmonella* Typhimurium Model Biofilms

Based on Figure 2B, it was observed that the viable cell density of the *S.* Typhimurium model biofilms significantly reduced following 10 min of CAP treatment. As for the *L. monocytogenes* model biofilms, similar results were previously obtained within the study of Govaert et al. (2019a). Nevertheless, when these CAP treated model biofilms were further incubated (with fresh growth medium and at the optimal biofilm formation temperature for *S.* Typhimurium), the viable cell density again became equal to the initial cell density of the untreated model biofilms. The effect of the CAP treatment was thus already nullified following 1 day of incubation due to the ability of the surviving biofilm-associated *S.* Typhimurium cells to continue multiplying. When the CAP treated biofilms were incubated for a longer period of time, no further change of the viable cell density was observed as compared to the CAP treated + 1 day incubated model biofilms. As for the *L. monocytogenes* model biofilms, this can again be an indication of the CAP treated + incubated biofilm-associated cells becoming more resistant (or less susceptible) toward environmental stress factors. Nevertheless, one should again be careful with generalizing the findings of the currently presented study, using a direct helium-based DBD system, since the applied treatment characteristics can have a significant influence on the ability of the CAP treated biofilm-associated cells to regrow.

A similar viable cell density trend was again observed for both growth media (Figure 2B). However, as for the *L. monocytogenes* model biofilms, generally lower values were always observed using the selective medium, indicating that sub-lethal injury of the cells occurred for each of the tested conditions. When comparing the percentages of sub-lethal injury, the highest value was observed for the CAP treated model biofilms. For this specific case, an average value of approximately 79% was obtained. Following further incubation of the CAP treated *S.* Typhimurium model biofilms, the percentage of sub-lethal injury (significantly) decreased. Nevertheless, these percentages remained generally higher than the corresponding value of approximately 44% which was observed for the untreated *S.* Typhimurium model biofilms. Therefore, it can be concluded that the highest risk of underestimating the level of contamination of food contact surfaces occurred directly following CAP treatment, although this risk remained relatively high for the CAP treated + incubated model biofilms.

With respect to the total biomass of the *S.* Typhimurium model biofilms (Figure 3B), no significant differences were observed between the untreated, CAP treated, and CAP treated + incubated samples. Based on this, it can be concluded that (i) CAP treatment did not result in (a significant) removal of the model biofilms and (ii) the surviving population of biofilm-associated *S.* Typhimurium cells was not able to continue producing matrix compounds. On the one hand, these results again confirm that the crystal violet staining assay is not sensitive enough to detect a significant increase of the viable cell density. On the other hand, the absence of an increase in biomass following further incubation of the CAP treated *S.* Typhimurium model biofilms does not necessarily mean that the resistance or susceptibility of the biofilm-associated cells toward any subsequent treatment method will remain the same. In the research of Govaert et al. (2018b), for example, the lack of an increased total biomass of the *S.* Typhimurium model biofilms (at an increased biofilm age) was still accompanied by an increased resistance toward CAP treatment. Therefore, further research is still required to examine this phenomenon in more detail.

Based on the results obtained for both biofilm forming species, it can be concluded that decontamination of food contact surfaces by means of a direct helium-based CAP treatment will require a properly designed cleaning schedule. With this respect, two important aspects need to be considered, i.e., (i) the efficacy of the CAP treatment needs to be further optimized, whether or not by combining it with another (mild) inactivation method, and (ii) further growth of the surviving population needs to be prevented. The former advice has been formulated based on the study of Ricker et al. (2018), which mentioned that regrowth of *P. aeruginosa* biofilms following thermal inactivation was not possible when the viable cell density was reduced to 10^3^ CFU/cm^2^. In future studies, it will be required to examine whether this minimal cell density is also valid for other biofilm forming species (including the two species examined within this research) and other inactivation methods such as CAP. The latter advice has been formulated based on the studies of Müsken et al. (2018) and Han et al. (2019), which mentioned that regrowth of biofilm-associated cells is strongly dependent on the encountered environmental conditions and the time in between two different treatments. Within the presented study, CAP treated model biofilms were incubated at the optimal biofilm formation conditions for both biofilm forming species. In future research, it needs to be examined whether regrowth of the biofilm-associated cells is also possible when these cells re-encounter less favorable environmental conditions, e.g., less nutrient-rich media (or no nutrients at all during equipment downtime) and lower incubation temperatures. In addition, regrowth of the biofilms could be prevented by limiting the time in between two surface decontamination steps, provided that the resistance/susceptibility of the biofilm-associated cells does not increase as the number of CAP treatments increases.

### Susceptibility of Biofilm-Associated Cells Toward Consecutive CAP Treatments

**Figure 4:**
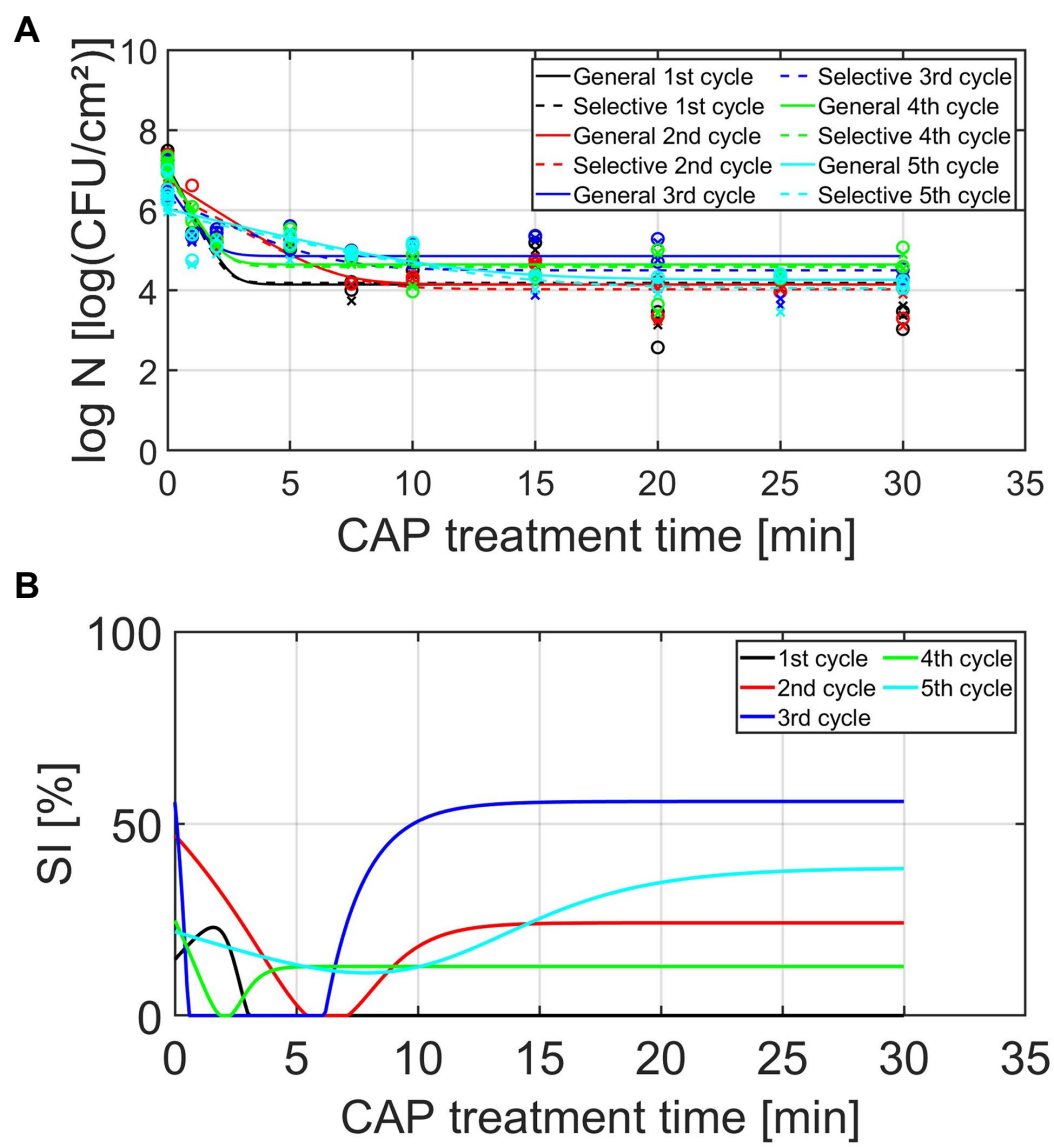
**(A)** Cell density [log_10_ (CFU/cm^2^)] and **(B)** percentage (%) of sub-lethally injured (SI) cells, both as function of the CAP treatment time for the *Listeria monocytogenes* model biofilms (*n* = 2). Five consecutive CAP treatment cycles were performed as: isolates from cycle × survivors were used to re-develop mature biofilms, which were again CAP treated (up to 30 min) in cycle x + 1. For the cell density, both the experimental data (symbols) and the global fit (line) of the Geeraerd et al. (2000) model are represented for each CAP treatment cycle: total viable population on non-selective medium (o, solid line) and uninjured viable population on selective medium (x, dashed line). For both the cell density and the percentage of SI, different CAP treatment cycles are indicated in different colors, i.e., black, red, blue, green, and light blue are used to illustrate the results obtained for the 1st, 2nd, 3rd, 4th, and 5th CAP treatment cycle, respectively.

**Figure 5:**
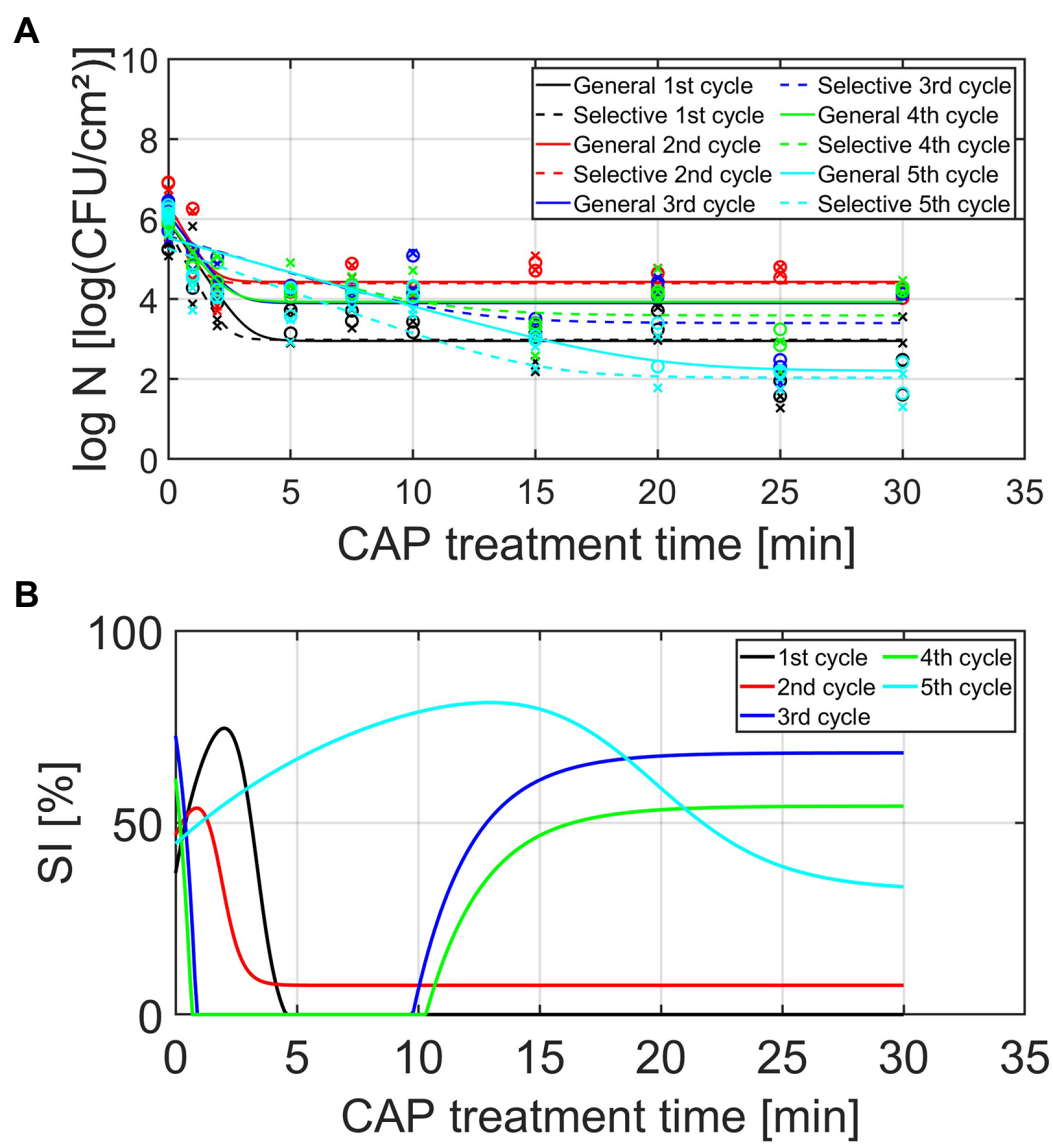
**(A)** Cell density [log_10_ (CFU/cm^2^)] and **(B)** percentage (%) of SI cells, both as function of the CAP treatment time for the *Salmonella* Typhimurium model biofilms (*n* = 2). Five consecutive CAP treatment cycles were performed as: isolates from cycle × survivors were used to re-develop mature biofilms, which were again CAP treated (up to 30 min) in cycle x + 1. For the cell density, both the experimental data (symbols) and the global fit (line) of the Geeraerd et al. (2000) model are represented for each CAP treatment cycle: total viable population on non-selective medium (o, solid line) and uninjured viable population on selective medium (x, dashed line). For both the cell density and the percentage of SI, different CAP treatment cycles are indicated in different colors, i.e., black, red, blue, green, and light blue are used to illustrate the results obtained for the 1st, 2nd, 3rd, 4th, and 5th CAP treatment cycle, respectively.

Based on the study of Govaert et al. (2019a), investigating the effect of different plasma characteristics on the inactivation efficacy of CAP for *L. monocytogenes* and *S*. Typhimurium model biofilms, it was concluded that none of the examined combinations resulted in complete inactivation of the biofilm-associated cells following an individual (direct) helium-based CAP treatment. A log-linear inactivation phase was always followed by a tail phase with a constant residual population. Based on the first part of this research, it can be assumed that this surviving population is able to further colonize the existing biofilm until a similar cell density is reached as for the untreated model biofilms. As mentioned before, some (small) parts of the biofilm or single cells might also detach from the biofilm, allowing the surviving biofilm-associated cells to form a biofilm elsewhere on the surface. For this purpose, it is of high importance to examine whether the biofilm-associated cells present within this newly developed biofilm can become more resistant or less susceptible toward CAP as the number of biofilm formation—CAP treatment cycles increases. Therefore, the viable cell density [log_10_ (CFU/cm^2^)] and the percentage (%) of sub-lethally injured cells have been determined as function of the CAP treatment time for five consecutive biofilm development—CAP treatment cycles. The results of these experiments have been illustrated in Figure 4 (*L. monocytogenes*) and Figure 5 (*S.* Typhimurium) and the corresponding model parameters of the Geeraerd et al. (2000) model have been presented in Table 1 (*L. monocytogenes*) and Table 2 (*S.* Typhimurium). The model parameters obtained using the non-selective medium will be discussed in detail in the following sections, while the corresponding selective medium model parameters will only be discussed in an indirect way by discussing the percentage of sub-lethally injured cells as function of the CAP treatment time.

**Table 1:** Model parameters obtained following CAP treatment of the *Listeria monocytogenes* model biofilms.

	Cycle 1	Cycle 2	Cycle 3	Cycle 4	Cycle 5
log_10_ *N_0_* non-selective medium [log(CFU/cm^2^)]	7.11 ± 0.27^c^	6.74 ± 0.18^b^	6.62 ± 0.18^b^	7.00 ± 0.18^c^	6.03 ± 0.15^a^
log_10_ *N_0_* selective medium [log(CFU/cm^2^)]	7.04 ± 0.21^e^	6.47 ± 0.20^c^	6.27 ± 0.20^b^	6.88 ± 0.18^d^	5.92 ± 0.16^a^
*kmax* non-selective medium (1/min)	2.65 ± 0.70^d^	0.89 ± 0.19^b^	2.14 ± 0.66^c^	2.38 ± 0.53^cd^	0.35 ± 0.09^a^
*kmax* selective medium (1/min)	2.74 ± 0.59^d^	0.76 ± 0.18^b^	0.62 ± 0.22^ab^	2.20 ± 0.52^c^	0.32 ± 0.09^a^
log_10_ *N_res_* non-selective medium [log(CFU/cm^2^)]	4.14 ± 0.19^a^	4.15 ± 0.16^a^	4.85 ± 0.12^c^	4.64 ± 0.12^b^	4.26 ± 0.18^a^
log_10_ *N_res_* selective medium [log(CFU/cm^2^)]	4.19 ± 0.15^b^	4.03 ± 0.18^a^	4.50 ± 0.18^c^	4.58 ± 0.12^c^	4.05 ± 0.21^ab^
log_10_-reduction non-selective medium [log(CFU/cm^2^)]	2.96 ± 0.33^d^	2.60 ± 0.24^c^	1.77 ± 0.21^a^	2.36 ± 0.22^b^	1.77 ± 0.24^a^
log_10_-reduction selective medium [log(CFU/cm^2^)]	2.85 ± 0.26^c^	2.44 ± 0.27^b^	1.77 ± 0.27^a^	2.30 ± 0.22^b^	1.87 ± 0.27^a^
RMSE non-selective medium (-)	0.6812	0.5097	0.4377	0.4512	0.4545
RMSE selective medium (-)	0.5390	0.5563	0.5470	0.4560	0.4886

For each CAP treatment cycle, the Geeraerd et al. (2000) model was used to fit the experimental data and to determine the initial cell density (log_10_ *N_0_*), the maximum inactivation rate (*k_max_*), the residual cell density (log_10_ *N_res_*), and the log_10_-reduction value. For each model fit, the corresponding Root Mean Squared Error (RMSE) value was provided as well. A separate ANOVA test was performed for each model parameter and each growth medium in order to determine whether an increased number of CAP treatment cycles resulted in significantly different model parameters. These significant differences have been indicated by means of a different letter, with “a” bearing the lowest value, and “b-e” bearing higher values per model parameter.

**Table 2:** Model parameters obtained following CAP treatment of the *Salmonella* Typhimurium model biofilms.

	Cycle 1	Cycle 2	Cycle 3	Cycle 4	Cycle 5
log_10_ *N_0_* non-selective medium [log(CFU/cm^2^)]	5.95 ± 0.27^b^	6.35 ± 0.16^d^	6.14 ± 0.25^c^	5.95 ± 0.17^b^	5.52 ± 0.21^a^
log_10_ *N_0_* selective medium [log(CFU/cm^2^)]	5.75 ± 0.31^c^	6.08 ± 0.21^d^	5.57 ± 0.26^bc^	5.54 ± 0.23^b^	5.26 ± 0.24^a^
*kmax* non-selective medium (1/min)	2.10 ± 0.65^bc^	2.44 ± 0.64^c^	1.96 ± 0.67^b^	1.88 ± 0.48^b^	0.39 ± 0.07^a^
*kmax* selective medium (1/min)	2.67 ± 0.85^b^	2.71 ± 1.11^b^	0.43 ± 0.15^a^	0.42 ± 0.15^a^	0.50 ± 0.11^a^
log_10_ *N_res_* non-selective medium [log(CFU/cm^2^)]	2.95 ± 0.19^b^	4.43 ± 0.11^d^	3.90 ± 0.17^c^	3.93 ± 0.11^c^	2.20 ± 0.35^a^
log_10_ *N_res_* selective medium [log(CFU/cm^2^)]	2.98 ± 0.21^b^	4.39 ± 0.13^d^	3.40 ± 0.30^c^	3.59 ± 0.26^c^	2.03 ± 0.31^a^
log_10_-reduction non-selective medium [log(CFU/cm^2^)]	3.00 ± 0.33^c^	1.93 ± 0.19^a^	2.24 ± 0.30^b^	2.02 ± 0.20^ab^	3.32 ± 0.40^d^
log_10_-reduction selective medium [log(CFU/cm^2^)]	2.77 ± 0.37^c^	1.69 ± 0.25^a^	2.17 ± 0.40^b^	1.95 ± 0.35^ab^	3.23 ± 0.40^d^
RMSE non-selective medium (-)	0.6917	0.4018	0.6215	0.4194	0.6651
RMSE selective medium (-)	0.7770	0.5102	0.7809	0.6889	0.7499

For each CAP treatment cycle, the Geeraerd et al. (2000) model was used to fit the experimental data and to determine the initial cell density (log_10_ *N_0_*), the maximum inactivation rate (*k_max_*), the residual cell density (log_10_ *N_res_*), and the log_10_-reduction value. For each model fit, the corresponding RMSE value was provided as well. A separate ANOVA test was performed for each model parameter and each growth medium in order to determine whether an increased number of CAP treatment cycles resulted in significantly different model parameters. These significant differences have been indicated by means of a different letter, with “a” bearing the lowest value, and “b-d” bearing higher values per model parameter.

Based on the graphs presented in Figures 4, 5, some general observations can be made independently from the biofilm forming species and the cycle number. Firstly, it can be observed that all inactivation curves had a similar shape, i.e., a log-linear inactivation phase was followed by a tail phase with a constant residual population. In other words, for each CAP treatment cycle, complete inactivation of the biofilm-associated cells was not obtained using currently applied (optimal) CAP treatment conditions. Secondly, it can be noticed that a certain percentage of the biofilm-associated cells was always sub-lethally injured prior to CAP treatment (i.e., at *t* = 0 min). A similar conclusion was drawn by Govaert et al. (2019a), where this phenomenon was deemed to be the result of the heterogeneous environmental conditions encountered within the three-dimensional biofilm structure. Finally, most CAP treatment cycles also resulted in a residual percentage of sub-lethally injured biofilm-associated cells. As mentioned before, these (relatively high) residual percentages can potentially result in an underestimation of the level of contamination, while using only selective media during the microbial assessment of food contact surfaces (Noriega et al., 2013).

#### *Listeria monocytogenes* Model Biofilms

Based on Table 1 and Figure 4, different observations can be done concerning the influence of the number of CAP treatment cycles on the *L. monocytogenes* biofilm inactivation kinetics. The initial cell density of the model biofilms (log_10_ *N_0_*) proved to be influenced by the number of CAP treatment cycles, i.e., this model parameter proved to decrease as the number of CAP treatment cycles increased. However, there was one exception, i.e., the log_10_ *N_0_* value obtained for the fourth cycle was similar to the corresponding value obtained for the first cycle. Based on this general trend, it can be concluded that the ability of the CAP treated cells to form biofilms with a similar cell density as for the untreated model biofilms (cycle 1, *t* = 0 min) appeared to decrease. The maximum inactivation rate (*k_max_*) also proved to be dependent on the specific CAP treatment cycle. Nevertheless, no clear correlation was observed between this model parameter and the number of CAP treatment cycles. For example, the low *k_max_* value obtained for cycle 2, while values for cycle 1 and 4 are higher, was unexpected. It should be stressed; however, that the lowest value was obtained for the fifth CAP treatment cycle, which indicates that the ability of the CAP species to penetrate into the biofilm matrix and/or their ability to interact with the biofilm-associated cells significantly decreased for this specific CAP treatment cycle. Previous biofilm studies using different antimicrobial agents proved that this limited penetration ability can be the result of an increased amount of EPS surrounding the biofilm-associated cells (e.g., Jiang et al., 2017). This can be highly disadvantageous as this might eventually reduce the CAP treatment efficacy. With respect to the residual cell density (log_10_ *N_res_*), the highest values were observed for the third and fourth CAP treatment cycle. In other words, the biofilm-associated cells proved to be more less susceptible to CAP when they were treated for the third and fourth time. For the fifth cycle, however, this susceptibility again became similar to the initial susceptibility of the biofilm-associated cells which were treated with CAP for the first time (i.e., cycle 1). Finally, it can be concluded that the efficacy of the CAP treatment (based on the log_10_-reduction values) also clearly depended on the number of CAP treatment cycles. When comparing these log_10_-reduction values, the following general trend was observed as: cycle 1 > cycle 2 > cycle 4 > cycle 3 = cycle 5. In other words, a decreasing trend can be observed for the efficacy of the CAP treatment as function of the number of CAP treatment cycles. However, as for the log *N_0_* values, an exception was again observed for the fourth cycle. Following five CAP treatment cycles, the log_10_-reduction value was on average 1.2 log_10_ (CFU/cm^2^) lower as compared to the first CAP treatment cycle. With respect to the percentage of sub-lethal injury as function of the CAP treatment time, it can be observed that both the initial and the residual percentage of sub-lethally injured cells were higher for CAP treatment cycles 2–5 as compared to CAP treatment cycle 1. Nevertheless, there was again no clear correlation between the specific cycle number and the sub-lethal injury values.

#### *Salmonella* Typhimurium Model Biofilms

Similar as for the *L. monocytogenes* model biofilms, different observations can be done regarding the influence of the number of CAP treatment cycles on the *S.* Typhimurium biofilm inactivation kinetics presented in Table 2 and Figure 5. As for the *L. monocytogenes* model biofilms, the initial cell density (log_10_ *N_0_*) proved to be influenced by the number of CAP treatment cycles. This model parameter first increased as the number of CAP treatment cycles increased, but this value afterward proved to decrease again. For the fifth cycle, this model parameter value became even lower than the corresponding value obtained for the first CAP treatment cycle. The ability of the cells to form biofilms with a similar cell density as for the untreated model biofilms (cycle 1, *t* = 0 min) was thus dependent on the specific CAP treatment cycle, which means that the initial bacterial load cannot be easily estimated. For the maximum inactivation rate (*k_max_*), only minor differences were observed. Only for the fifth CAP treatment cycle, significantly lower values were obtained as compared to the first CAP treatment cycle. This reduced inactivation rate might again be related to the ability of the CAP species to penetrate inside the biofilm matrix and/or to interact with the biofilm-associated cells. However, this phenomenon needs to be examined in more detail in order to draw any firm conclusions. With respect to the residual cell density values (log_10_ *N_res_*), the following general trend was observed as: cycle 2 > cycle 4 = cycle 3 > cycle 1 > cycle 5. The susceptibility of the biofilm-associated cells first decreased and then increased at an increased number of CAP treatment cycles. Moreover, the susceptibility of the biofilm-associated *S.* Typhimurium cells was even higher for the fifth cycle than for the first cycle. For the log_10_-reduction values, the efficacy of the CAP treatment first proved to decrease at an increased number of CAP treatment cycles, but the efficacy afterward increased again to higher values for the fifth cycle than for the first cycle. For the *L. monocytogenes* model biofilms, the opposite trend was observed, i.e., the efficacy proved to decrease for the fifth CAP treatment cycle. With respect to the percentages of sub-lethal injury as function of the CAP treatment time, it can be concluded that both the initial and the residual percentages observed for CAP treatment cycles 2–5 were (on average) higher than the corresponding values obtained for CAP treatment cycle 1. However, as for *L. monocytogenes*, no clear correction was observed between the specific CAP treatment cycle and the amount of sub-lethally injured cells.

As mentioned in the introduction, no previous studies examined the effect of an increased number of consecutive biofilm development—CAP treatment cycles on the susceptibility (or resistance) of biofilms toward CAP. However, similar investigations have been performed for treatment of clinical biofilms with certain antibiotics. The research of Ahmed et al. (2018), for example, exposed *P. aeruginosa* biofilms to sub-inhibitory concentrations of Ciprofloxacin. The surviving biofilm-associated cells were recovered and further used to develop new biofilms which were again treated with Ciprofloxacin at the same sub-inhibitory concentration. The percentage of resistant cells was quantified following each treatment step (six in total). The results indicated that this percentage of resistant cells significantly increased as the number of consecutive treatment steps increased. However, there was some fluctuation, i.e., there was no clear trend between the specific treatment step and the percentage of resistant cells (Ahmed et al., 2018). Similar observations were made within the presented study, i.e., there was often no clear/linear correlation between the specific CAP treatment cycle and the model parameter values. Within the study of Ahmed et al. (2018), this was deemed to be a consequence of competition between clones with a different fitness cost. In order to examine this in more detail, future studies should examine whether phenotypical and/or genotypical changes occur as a result of the consecutive CAP treatment cycles. For the phenotypical characterization of the cells, Phenotype Microarrays could be used to assess, among others, the ability of the (un)treated biofilm-associated cells to use different nutrient sources (Bochner, 2009). In addition, it should be examined as well whether similar conclusions can be drawn, while using an air-based and/or indirect plasma system since these CAP characteristics have a high influence on the specifically generated reactive plasma species and their ability to interact with the biofilm-associated cells.

## Conclusion

The results of this study proved that incomplete inactivation of the *L. monocytogenes* and *S.* Typhimurium model biofilms resulted in regrowth of the biofilm-associated cells, i.e., the surviving population of CAP treated biofilm-associated cells was able to continue multiplying (*L. monocytogenes* and *S.* Typhimurium) and producing an increased amount of EPS (*L. monocytogenes*). Moreover, isolates of these surviving biofilm-associated cells were also able to form new biofilms with an altered susceptibility toward the (direct) helium-based CAP treatment. In general, the CAP treatment efficacy proved to decrease at an increased number of biofilm formation—CAP treatment cycles. A detailed understanding of the behavior of the CAP surviving population will help to decide whether the CAP technology can be safely implemented in the food industry. Both phenomena can result in increased health risks as this might result in an overestimation of the CAP treatment efficacy and, consequently, a higher chance of contamination of food products. Therefore, additional measures should be taken in order to (i) prevent regrowth of the CAP treated model biofilms and (ii) the formation of (highly resistant or less susceptible) biofilms elsewhere on the surface. The surviving population of biofilm-associated cells needs to be further reduced, which can potentially be obtained by combining the CAP technology with another (mild) inactivation technology. If this combined treatment cannot result in complete inactivation of the biofilm-associates cells, the time in between two treatment cycles for surface decontamination should be limited in order to prevent further colonization of the existing biofilms and the development of new biofilms. In addition, the environmental conditions (e.g., surface temperature) should be selected as such that biofilm formation is inhibited.

## Data Availability Statement

The raw data supporting the conclusions of this article will be made available by the authors, without undue reservation.

## Author Contributions

MG, CS, and JV: conceptualization and supervision. MG and CS: methodology, validation, and formal analysis. MG: software, data curation, writing—original draft preparation, visualization, and project administration. MG and CA: investigation. JW and JV: resources. MG, CS, JW, and JV: writing—review and editing. JV: funding acquisition. All authors contributed to the article and approved the submitted version.

## Funding

This research was funded by the KU Leuven Research Council, project C24/18/046 and the Fund for Scientific Research-Flanders, project G.0B41.21N and SBO project S.0081.21N.

## Conflict of Interest

The authors declare that the research was conducted in the absence of any commercial or financial relationships that could be construed as a potential conflict of interest.

## Publisher’s Note

All claims expressed in this article are solely those of the authors and do not necessarily represent those of their affiliated organizations, or those of the publisher, the editors and the reviewers. Any product that may be evaluated in this article, or claim that may be made by its manufacturer, is not guaranteed or endorsed by the publisher.
